# Blocked at the Stomatal Gate, a Key Step of Wheat *Stb16q*-Mediated Resistance to *Zymoseptoria tritici*

**DOI:** 10.3389/fpls.2022.921074

**Published:** 2022-06-27

**Authors:** Mélissa Battache, Marc-Henri Lebrun, Kaori Sakai, Olivier Soudière, Florence Cambon, Thierry Langin, Cyrille Saintenac

**Affiliations:** ^1^Université Clermont Auvergne, INRAE, GDEC, Clermont-Ferrand, France; ^2^Université Paris-Saclay, INRAE, UR BIOGER, Thiverval-Grignon, France

**Keywords:** Septoria tritici blotch, *Stb16q*, resistance, avirulence, leaf penetration, stomata

## Abstract

Septoria tritici blotch (STB), caused by the fungus *Zymoseptoria tritici*, is among the most threatening wheat diseases in Europe. Genetic resistance remains one of the main environmentally sustainable strategies to efficiently control STB. However, the molecular and physiological mechanisms underlying resistance are still unknown, limiting the implementation of knowledge-driven management strategies. Among the 22 known major resistance genes (*Stb*), the recently cloned *Stb16q* gene encodes a cysteine-rich receptor-like kinase conferring a full broad-spectrum resistance against *Z. tritici*. Here, we showed that an avirulent *Z. tritici* inoculated on *Stb16q* quasi near isogenic lines (NILs) either by infiltration into leaf tissues or by brush inoculation of wounded tissues partially bypasses *Stb16q*-mediated resistance. To understand this bypass, we monitored the infection of GFP-labeled avirulent and virulent isolates on *Stb16q* NILs, from germination to pycnidia formation. This quantitative cytological analysis revealed that 95% of the penetration attempts were unsuccessful in the *Stb16q* incompatible interaction, while almost all succeeded in compatible interactions. Infectious hyphae resulting from the few successful penetration events in the *Stb16q* incompatible interaction were arrested in the sub-stomatal cavity of the primary-infected stomata. These results indicate that *Stb16q*-mediated resistance mainly blocks the avirulent isolate during its stomatal penetration into wheat tissue. Analyses of stomatal aperture of the *Stb16q* NILs during infection revealed that *Stb16q* triggers a temporary stomatal closure in response to an avirulent isolate. Finally, we showed that infiltrating avirulent isolates into leaves of the *Stb6* and *Stb9* NILs also partially bypasses resistances, suggesting that arrest during stomatal penetration might be a common major mechanism for *Stb*-mediated resistances.

## Introduction

*Zymoseptoria tritici* (telemorph *Mycosphaerella graminicola*) is the causal agent of Septoria tritici blotch (STB), one of the most devastating diseases of wheat in Europe. This fungal pathogen causes, on average, 10% of annual yield losses, representing a value of more than $800 (€720) million despite the use of fungicides and resistant wheat varieties (Fones and Gurr, 2015; Torriani et al., 2015). Because of its economic importance, the infection biology of this fungus has been extensively studied (Cohen and Eyal, 1993; Kema, 1996; Duncan and Howard, 2000; Rohel et al., 2001; Shetty et al., 2003; Keon et al., 2007; Jing et al., 2008; Siah et al., 2010; Fones et al., 2017; Haueisen et al., 2019; Fantozzi et al., 2021). Under high-humidity conditions, *Z. tritici* spores present on the leaf surface germinate. The resulting hyphae initiate pathogenesis by developing epiphytically before penetrating wheat leaf mainly through stomata. After penetration, inner infectious hyphae colonize the leaf apoplast. This extended invasive phase is defined as the asymptomatic phase. Usually, after 7–14 days post infection (dpi) in a growth chamber, chlorotic and necrotic lesions appear and announce the beginning of the symptomatic or necrotrophic phase. This phase is associated with the formation of pycnidia in sub-stomatal cavities that finally release pycnidiospores through the ostiole to initiate another infection cycle.

Genetic resistance is the main environmentally sustainable and efficient strategy to control STB. Important advances in the genetic architecture of wheat resistances against *Z. tritici* have been made these past few years, with the mapping of 22 major *Stb* genes and more than 167 quantitative trait loci (QTLs) spread throughout all chromosomes (Brown et al., 2015; Yang et al., 2018). The *Stb* genes have been very attractive for wheat breeding as they confer strong resistances inherited from a single locus. However, the rapid emergence of virulent isolates considerably limits their durability, as illustrated by the rapid breakdown of most *Stb*-mediated resistances largely deployed in the fields (McDonald and Linde, 2002; Brown et al., 2015). Functional characterization of the resistance mechanisms controlled by the *Stb* genes is now needed for breeding efficient and durable wheat resistances to STB.

During interactions between *Z. tritici* and resistant wheats, little is known about when, where, and how non-pathogenic isolates are stopped. In previous comparative cytological studies between compatible and incompatible interactions, fungal growth was reported to be stopped after penetration through stomata, at different steps during resistant wheat apoplast colonization (Cohen and Eyal, 1993; Kema, 1996; Shetty et al., 2003; Jing et al., 2008; Siah et al., 2010). No hypersensitive responses (HR) and no lignin or polyphenolic depositions were observed (Cohen and Eyal, 1993; Kema, 1996). Cellular autofluorescence (Cohen and Eyal, 1993; Duncan and Howard, 2000; Shetty et al., 2003), ROS, and callose accumulation (Cohen and Eyal, 1993; Shetty et al., 2003) have been reported, but their roles in resistance are unknown. Comparative proteomic (Yang et al., 2015) and metabolomic (Seybold et al., 2020) analyses revealed an early upregulation of carbohydrate metabolism, cell wall reinforcement, and the production of defense proteins and possible antifungal metabolites generating an apoplastic environment stressful for the fungus. In all these studies, exploration of wheat features linked to resistance relied on the comparison between distinct genetic backgrounds for which the underlying genetic determinism of resistance is unknown. Thus, assignment of molecular or physiological events to *Stb* genes or QTLs is not possible, which strongly limits our understanding of the nature, diversity, and interaction of resistance mechanisms against STB.

The recent cloning of the *Stb6* and *Stb16q* genes (Saintenac et al., 2018, 2021) is a valuable resource to fast forward search for *Stb*-mediated resistance mechanisms. Both *Stb* genes encode receptor-like kinases (RLKs), which, in plant immunity, are thought to recognize invasion patterns in the apoplast and transduce a defense signal (van der Burgh and Joosten, 2019). *Stb6* controls a qualitative gene-for-gene resistance against *Z. tritici* isolates carrying the matching *AvrStb6* and encodes for a wall-associated kinase (WAK) (Kema et al., 2000; Brading et al., 2002; Saintenac et al., 2018). Many WAKs monitor plant cell wall integrity and induce a broad range of host defense responses, including the activation of MAPK cascades, the modulation of hormones signaling, and the modification of cell wall composition to prevent pathogen penetration (Stephens et al., 2022). *Stb16q*, of particular interest as it confers qualitative broad-spectrum resistance against a large panel of *Z. tritici* isolates, encodes a cysteine-rich-receptor-like kinase (CRK) (Saintenac et al., 2021). Most of the functional analyses of this RLK family arise from studies on *Arabidopsis thaliana*, wherein CRKs were shown to be involved in ROS signaling, modification of phytohormonal pathways, cell death, and/or stomatal immunity (Acharya et al., 2007; Bourdais et al., 2015; Yeh et al., 2015; Lee et al., 2017; Yadeta et al., 2017; Kimura et al., 2020).

This study aimed at deciphering the mechanisms involved in *Stb16q*-mediated resistance. We performed a quantitative phenotyping analysis between compatible and incompatible interactions by brush-inoculating leaves. We also evaluated the impact of inoculating spores either by infiltration into leaf or by brush-inoculation on wounded tissues on *Stb16q*-mediated resistance. We also questioned the effect of these inoculation methods on *Stb6*- and *Stb9*-mediated resistances. Using a comparative quantitative cytological approach, we investigated when and where an avirulent isolate was stopped during *Stb16q*-mediated resistance. Finally, we tested whether stomatal immunity is involved in *Stb16q*-mediated resistance using cytological and biochemical approaches.

## Materials and Methods

### Plant and Fungal Materials

All experiments were performed using wheat Chinese Spring (CS) near isogenic lines (NILs), carrying either no S*tb* gene (named NIL^stb^), or one of the following *Stb* genes *Stb6*, *Stb9*, and *Stb16q* and named NIL^stb^, NIL^Stb6^, NIL^Stb9^, NIL^Stb16q^, respectively. NIL^stb^ and NIL^Stb6^ were obtained following five backcrosses with the recurrent parent CS starting with F_1_ CS × Courtot. At each generation, plants were phenotyped with *Z. tritici* isolate IPO323 and genotyped with SSR marker gwm369 and *Stb6* diagnostic SNP markers to maintain *Stb6* heterozygosity in the progenies. A BC_5_F_1_ plant heterozygous for *Stb6* was self-fertilized. A BC_5_F_2_ carrying the resistance *Stb6* haplotype 1 and a BC_5_F_2_ carrying the susceptible *Stb6* haplotype 3 at the homozygous state were selected and named NIL^Stb6^ and NIL^stb^, respectively. NIL^Stb9^ was developed by performing five backcrosses, with the recurrent parent CS starting with F_1_ CS × Courtot. At each generation, the plants were phenotyped with *Z. tritici* isolates IPO89011 and genotyped with SSR markers barc129 and wmc317 to maintain *Stb9* heterozygosity in the progenies. A BC_5_F_1_ plant heterozygous for *Stb9* was crossed with NIL^stb^, and a progeny heterozygous for *Stb6* and *Stb9* was self-fertilized. A progeny carrying the resistance allele of *Stb9* and the susceptible allele of *Stb6* both at the homozygous state was selected and named NIL^Stb9^. The NIL^Stb16q^ was developed similarly to NIL^Stb9^ by starting with F1 CS × TA4152-19 and by using IPO9415 and *Stb16q* diagnostic markers cfn80044 and cfn80045 (Saintenac et al., 2021).

*Z. tritici* isolates IPO9415 (avirulent on *Stb16q* and virulent on *Stb6* and *Stb9*) and CFZ008 (virulent on *Stb6*, *Stb9*, and *Stb16q*) were collected in French wheat fields on cultivar Premio in 2009 and cultivar Cellule in 2016, respectively. The *Stb9*-avirulent IPO89011 isolate (avirulent on *Stb16q*) was collected from a Netherlands wheat field (Brown et al., 2001). The *Stb6*-avirulent IPO323 isolate (avirulent on *Stb16q*), collected on cultivar “Arminda” in the Netherlands in 1981, is the genome reference isolate (Goodwin et al., 2011). For cytological analyses, plasmid pYSKH-4 was introduced into *Z. tritici* isolates IPO9415 and CFZ008 using *Agrobacterium tumefasciens*-mediated transformation (ATMT) according to published protocols (Marchegiani et al., 2015). Plasmid pYSKH-4 contains eGFP expressed under the control of a strong constitutive promoter TEF1 and the ILV1-R gene, conferring resistance to sulfonylurea (Sidhu et al., 2015). Transformants resistant to sulfonylurea were purified by monospore isolation, and their GFP fluorescence was assessed as previously described (Marchegiani et al., 2015).

### Plant and *Zymoseptoria tritici* Growing Conditions

Except for cytological analyses, all experiments were performed using the attached leaf assay (Lee et al., 2015b). The plants were grown in 60 cm × 40 cm trays filled with 1/2 blond and 1/2 brown peat mosses (Humustar soil, NPK 14-16-18, SARL Activert, Riom, France) in the MTR30 growth chamber (Conviron^®^) equipped with fluorescent tubes (Master TL-D Super 80 70 W/840; 480 mol. m^–2^.s^–1^; Philips, Amsterdam, the Netherlands) under a 16-h photoperiod at 21/18°C (day/night) and 80% relative humidity (RH). For cytological analyses, the plants were grown in a 10 cm × 10 cm pot filled with the Floradur^®^ B soil (NPK 14-16-18; Floragard Vertriebs-GmbH, Oldenburg, Germany) in a controlled growth chamber with fluorescent tubes (Osram Lumilux L58W/830; 300 mol. m^–2^. s^–1^; OSRAM GmbH, Munich, Germany), under a 16-h photoperiod at 22/18°C (day/night) and 80% RH.

*Z. tritici* isolates were grown in liquid YG with 100 mg/L streptomycin and 100 mg/L ampicillin at 20°C, 180 rpm, for 3 days and spread on YPD plates supplemented with the same antibiotics at 20°C for 4 days. A suspension of 1 × 10^6^ spores/ml supplemented with 0.05% (v/v) Tween-20 was prepared to inoculate attached leaves. For cytological analyses, *Z. tritici* isolates IPO9415-GFP and CFZ008-GFP were grown on YPD plates supplemented with 100 mg/L ampicillin at 18°C, 70–80% RH for 3 days, and spread again on new YPD plates for 4 more days. A suspension of 3 × 10^6^ spores/ml supplemented with 10% (v/v) gelatine was prepared to inoculate unattached leaves.

### Inoculation Procedures

Six- to eight-centimeter sections of the second leaf of 14-day-old NILs were inoculated with a paintbrush six times repeated twice (or 3 times twice for cytological analyses) with spore suspensions or water supplemented with 0.05% (v/v) Tween-20 as control solution. Infiltration assays were performed by infiltrating between 0.1 and 0.5 ml of spores suspensions or control solution at three different locations in second leaves using a needle-less syringe. The wounding assay was conducted by gently scrapping 3 times the leaf surface using waterproof abrasive paper (240 grit) before brush-inoculation. Following inoculation, the plants were covered with transparent bags for 3 days before returning to normal conditions, except for the 100% RH experiment where the bags were maintained for 7 days. Disease severity of inoculated leaves was visually evaluated every 2 days from 8 to 21 dpi by estimating the percentage of the leaf surface covered with symptoms (chlorosis and necrosis) and pycnidia. Results were obtained from three to eight individual leaves per condition from one to two independent experiments. The area under disease progression curve (AUDPC) for symptoms and pycnidia was calculated using the “audpc” function of the “agricolae” package in R software (version 4.1.0).

### Quantification of Fungal Biomass

The inoculated leaves were harvested 10 h after the light turned on every day from 0 to 10 days post inoculation (dpi) and at 14 dpi. The collected leaves were snapfrozen and stored at –80°C. Total DNA was isolated using the CTAB method (Fabre et al., 2019), treated with 10 mg/ml RNase A (Sigma-Aldrich) at 37°C for 1 h and quantified using the Hoechst 33258 method (Thermo Fisher Scientific), with the Tecan’s Infinite M1000 microplate reader. Real-time quantitative PCR was performed with the LightCycler^®^ 480 SYBR Green I Master Mix (Roche) on 25 ng total DNA in a final volume of 15 μL, using the *Z. tritici* IGS primers (Forward: 5′-CGACGGCGTATCGTAATTT-3′/Reverse: 5′-CAACAAATCGAGCCGACGT-3′) to monitor fungal biomass and the wheat 18S primers (Forward: 5′-CCATCCCTCCGTAGTTAGCTTCT-3′/Reverse: 5′-CCTGTCG GCCAAGGCTATATAC-3′) to monitor plant biomass. Reactions were run in triplicate with the following thermal cycling profile: 95°C for 10 min, followed by 45 cycles of 10 s at 95°C, 15 s at 60°C, and 15 s at 72°C and completed with a melting curve analysis program. The relative expression was calculated with the 2^–ΔΔ*Ct*^ method (Livak and Schmittgen, 2001). Results were obtained from ten individual leaves per condition from two independent experiments.

### Sporulation Assay

The sporulation assay was performed as described previously (Lee et al., 2015a) with few modifications. At 24 dpi, 6-cm sections of inoculated leaves were excised and placed individually in sterile 15-ml Falcon tubes containing compresses saturated with water. The tubes were incubated at 15°C for 48 h in the dark. Two milliliters of water supplemented with 0.05% Tween 20 were added in each tube, which were then vortexed for 30 s to wash pycnidiospores off the leaves. The spores were counted using the Malassez counting chamber. Results were obtained from nine individual leaves per condition from two independent experiments.

### Cytological Analysis

Two-centimeter sections of infected leaves were harvested at 2, 4, 6, 9, and 13 dpi. These sections were stained 30 s with 0.1% Calcofluor White M2R (Sigma-Aldrich) in water, briefly rinsed in water, set on slides with double-side adhesive tape and mounted in Perfluorodecalin (Sigma-Aldrich). Stained samples were observed using a Leica DM5500 B fluorescent microscope with GFP (ex: 450–490 nm, em: 500–550 nm) and UV filters (ex: BP, 340–380 nm, em: LP, 425 nm) to visualize the GFP transgenic fungal line and Calcofluor White M2R, respectively. Five random Z-stack images of 621 μm × 466 μm for each 2-cm leaf sections were acquired under a 20×/0.5 dry objective using the LAS AF software (version 3.2.0.9652). Images were then analyzed for quantification of the spores germination rate (number of germinated spores relative to the total number of spores), the percentage of stomata reached by epiphytic hyphae (number of stomata in contact with at least one hypha relative to the total number of stomata), the percentage of penetration attempts (number of stomata with hyphae extremity, often with appressoria-like structures, diving into its ostiole relative to the total number of stomata), the percentage of successful penetrations events (number of stomata with a penetration attempt on their ostiole and with infectious hyphae in their sub-stomatal cavities relative to the total number of stomata), the percentage of secondary sub-stomatal cavities colonization (number of stomata with hyphae only in the sub-stomatal cavity, without hyphae above the ostiole relative to the total number of stomata), the percentage of early-stage pycnidia (number of stomata with branching and ring-forming hyphae in the sub-stomatal cavity relative to the total number of stomata), and the percentage of young and mature pycnidia (number of stomata with a densified hyphal structure, in the form of GFP halo, relative to the total number of stomata) (Supplementary Figure 3). The remainder of stained-infected leaves was mounted in Perfluodecalin and was observed with a Leica SPE confocal under a 40X/1.15 APO OIL objective. Samples were illuminated sequentially with 488 nm (detection range, 500–554 nm) and 405 nm (detection range, 436–475 nm) lasers for GFP and CalcoFluor White M2R visualization, respectively. Images were acquired with the LAS AF software (version 3.1.3). Results were obtained from six individual leaves per condition from two independent experiments.

### Stomatal Aperture Assays

Imprints of leaves were made using the Aquasil^®^ Ultra + LV DENTSPLY SIRONA dental resin (Henry Schein). Transparent nail polish was applied on imprints, transferred on slides, and observed using an Axio Observer Z1 (Zeiss) microscope, with phase contrast under a × 16 magnification. Tiles images of 0.5 cm^2^ were acquired with Zeiss Zen 3.1 software (Blue edition) and then analyzed with the Fiji package of ImageJ (version 2.3.0). Stomatal aperture was then evaluated differently according to the different assays. To study stomatal behavior during 3 consecutive days, 3 tiles images per leaf were acquired, and the ratio ostioles areas over stomata areas of 50 stomata was measured for each tile images using the “freehand” tool of Fiji. Results were obtained from two individual leaves at each time point from one experiment. To observe the impact of *Stb16q* on stomatal opening, imprints were made at midday. One experiment was conducted in a controlled MTR30 Conviron^®^ growth chamber as described above, and one in a growth chamber with a halogen lightening (Powerstar HQI-TS 250W/D Pro; 250 mol. m^–2^.s^–1^; OSRAM GmbH, Munich, Germany). To artificially open stomata, 4 μM fusicoccin (Sigma-Aldrich) or 4 μM coronatine (Sigma-Aldrich) prepared in water supplemented with 4% (v/v) DMSO was sprayed at midday evenly on second leaves using a micro-diffuser sprayer (Ecospray). Imprints were made 30 min, 2 h or 4 h post application. To evaluate *Stb16q* and toxins impact on stomatal opening, one tile image per leaf was acquired, and all unblurred stomata on each image were classified into “closed” or “opened” categories using the “cell counter” plugin of Fiji. Results were obtained from three to five independent leaves per condition from one experiment.

### Statistical Analyses

Statistical analyses were carried out using the R software version 4.1.0. Data are expressed as mean ± standard error of the mean. The differences in fungal biomass were analyzed using a linear mixed model with a Tukey’s multiple range test. To evaluate the impact of *Stb16q* on stomatal aperture, proportions of closed stomata were analyzed for each day post inoculation independently using a linear mixed model. Analysis of cytological data was performed using the one-way non-parametric Van der Waerden test from “agricolae” package, combining wheat genotype, day post inoculation, and treatment in a single factor. Spores concentrations, AUDPC, and the proportions of closed stomata following 100% RH or toxin application were analyzed using the non-parametric multi-factorial method Aligned Rank Transformation (ART) ANOVA with the ART function of the ARTool package. All *p*-values < 0.05 were considered to be significant.

## Results

### *Stb16q*-Mediated Resistance Establishes During the Asymptomatic Phase

**FIGURE 1:**
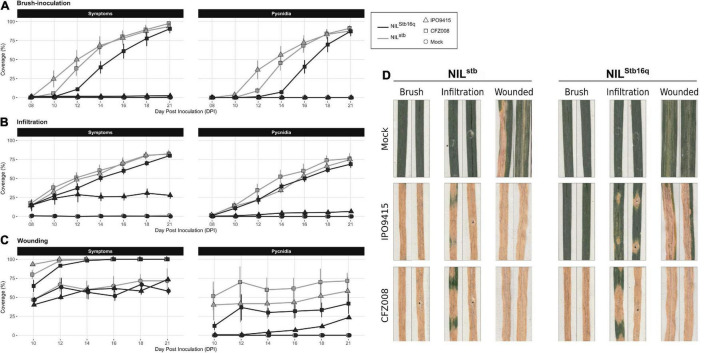
Evolution of symptoms (chlorosis + necrosis) and regions bearing pycnidia of NIL^Stb16q^ (black) and NIL^stb^ (gray) inoculated with control solution [water/Tween-20, 0.05% (v/v); = Mock, circles], the CFZ008 virulent (squares) or the IPO9415 avirulent (triangles) *Z. tritici* isolates using brush-inoculation **(A)**, leaf infiltration **(B)** or brush-inoculation after wounding **(C)**. **(D)** Images of two representative second leaves at 21 dpi. Values are means ± SEM [*n* = 8 for **(A,B)**; *n* = 3 for **(C)**].

*Stb16q* wheat quasi near-isogenic lines (NIL^Stb16q^ and NIL^stb^) were inoculated with the *Stb16q*-avirulent IPO9415 and the *Stb16q*-virulent CFZ008 isolates using the brush-inoculation method, which mimics natural infection. In the compatible IPO9415/NIL^stb^ interaction, symptoms and pycnidia appeared at 10 and 12 dpi, respectively. Two- and 4-day delays in symptoms emergence were observed for isolate CFZ008 on NIL^stb^ and NIL^Stb16q^, respectively, compared to the IPO9415/NIL^stb^ interaction. For all three compatible interactions, the leaf surface covered with symptoms and pycnidia gradually increased until almost full coverage at 21 dpi (Figures 1A,D). By contrast, no symptoms were observed in the incompatible IPO9415/NIL^Stb16q^, except rare chlorosis (< 3% of the leaf surface). Moreover, pycnidia were not observed at any time during this interaction, and pycnidiospores could not be detected in our sporulation induction assay (Supplementary Figure 1B). In addition, the growth of the IPO9415 isolate on both *Stb16q* NILs was monitored using real-time qPCR. Until 6 dpi, no significant increase in fungal biomass was observed neither in compatible nor in incompatible interactions (Supplementary Figure 1A). Then, fungal biomass increased by a factor 2–3 every day from 7 to 14 dpi in the compatible interaction, while it remained unchanged in the incompatible interaction. These results showed that *Stb16q*-mediated resistance establishes before 7 dpi, during the asymptomatic phase.

### Infiltration and Wounding Allow Avirulent *Zymoseptoria tritici* to Partially Bypass *Stb16q*-Mediated Resistance

In parallel to brush-inoculation, artificial infiltration of spores of isolates IPO9415 and CFZ008 directly into the apoplast of *Stb16q* NILs was performed. Symptoms and pycnidia appeared as soon as 8 and 10 dpi, respectively, simultaneously in the three compatible interactions, and gradually extended until reaching 81% ± 1% and 73% ± 4%, respectively, of leaf coverage at 21 dpi (Figures 1B,D). Despite a delay in symptoms emergence between infiltration and brush-inoculation, no significant differences in the area under disease progression curve (AUDPC) were observed (Supplementary Figure 2A). Interestingly, in the incompatible IPO9415/NIL^Stb16q^ interaction, symptoms and pycnidia were observed at 8 and 12 dpi, respectively (Figure 1B). However, in the incompatible interaction, the leaf surface covered with symptoms and pycnidia did not extend over 28 and 7%, respectively, at 21 dpi (Figure 1D and Supplementary Figure 2A). Isolates IPO9415 and CFZ008 were also inoculated on *Stb16q* NILs leaves gently wounded with abrasive paper. Wounding alone (mock) induced chlorosis and necrosis on leaves of both NILs (Figures 1C,D and Supplementary Figure 2B). In the three compatible interactions, pycnidia were observed at 10 dpi with 35% ± 20% of leaf coverage, and extended up to 57% ± 15% by 21 dpi (Figures 1C,D). In the incompatible interaction, pycnidia appeared at 14 dpi and covered 23% of the leaves at 21 dpi (Figures 1C,D and Supplementary Figure 2B). Together, these results showed that *Stb16q*-mediated resistance is partially bypassed when using inoculation methods (wounding and infiltration) that circumvent the penetration process, suggesting that this resistance essentially operates during *Z. tritici* penetration into a wheat leaf.

### *Stb16q*-Mediated Resistance Arrests Avirulent *Zymoseptoria tritici* During Stomata Penetration

To investigate whether *Stb16q* stops *Z. tritici* during its penetration into leaves, the infection processes of GFP-labeled IPO9415 and CFZ008 isolates inoculated on *Stb16q* NILs were quantitatively monitored using epifluorescence and confocal microscopies (Supplementary Table 1). Inoculated leaves were stained with the fungal chitin dye calcofluor to distinguish between epiphytic hyphae located on the leaf surface (both GFP-fluorescent and calcofluor-stained) and infectious hyphae located inside the leaf (only GFP fluorescent).

**FIGURE 2:**
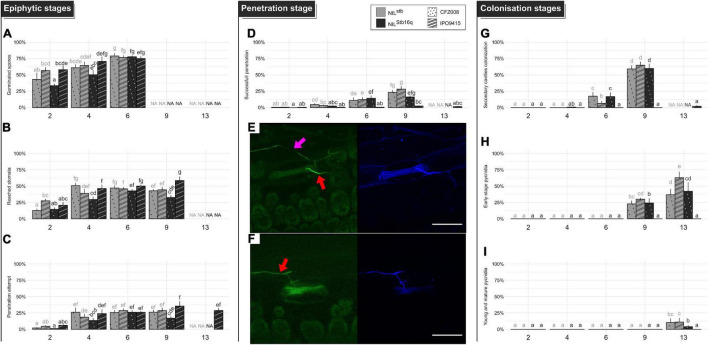
Quantitative cytological assessment of the different stages of infection of the CFZ008 virulent (dotted bars) or the IPO9415 avirulent (stripped bars) *Z. tritici* GFP-labeled isolates inoculated on NIL^Stb16q^ (black bars) and NIL^stb^ (gray bars). **(A)** Germination (= number of germinated spores relative to the total number of spores). **(B)** Reached stomata (= number of stomata with hyphae in contact relative to the total number of stomata). **(C)** A penetration attempt (= number of stomata with hyphae diving into the ostiole relative to the total number of stomata). **(D)** Successful penetration (= number of stomata with hyphae going through the ostiole and in the sub-stomatal cavity relative to the total number of stomata). **(G)** Secondary sub-stomatal cavities colonization (= number of stomata with hyphae only in the sub-stomatal cavity, without hyphae above the ostiole, relative to the total number of stomata). **(H)** Early-stage pycnidia (= number of stomata with branching and ring-forming hyphae in the sub-stomatal cavity relative to the total number of stomata). **(I)** Young and mature pycnidia (= number of stomata with densified hyphae structure, in the form of the GFP halo, relative to the total number of stomata). Values are means ± SEM (*n* = 6). Different letters indicate significantly different values (Van der Waerden test, *p* < 0.05). Representative confocal images of penetration events at 6 dpi for NIL^stb^ **(E)** and NIL^Stb16q^ **(F)**, inoculated with the GFP-labeled IPO9415 isolate (green) and stained with the fungal surface-dye calcofluor (blue). Red and pink arrows indicate hyphae on the leaf surface (green and blue) and internal hyphae (only green), respectively. Bar = 50 μm.

To assess the epiphytic phase, we calculated (i) the spore germination rate (Supplementary Figure 3A), (ii) the percentage of stomata reached by epiphytic hyphae (Supplementary Figure 3B), and (iii) the percentage of penetration attempts (Supplementary Figure 3C). In the three compatible interactions, approximatively half (44% ± 12%) of the spores germinated at 2 dpi, reaching 78% ± 1% by 6 dpi (Figure 2A). It was not possible to assess the spore germination rate after 6 dpi, as too many epiphytic hyphae were present on the leaf surface. By 2 dpi, epiphytic hyphae have already reached 18% ± 8% of stomata, and, by 4 dpi, they were in contact with 40% ± 11% of stomata (Figure 2B). Among these reached stomata, half (which represented 19% ± 7% of total stomata) underwent a penetration attempt by 4 dpi (Figure 2C), which was frequently associated with fungal appressoria-like structures located on stomata ostiole. The epiphytic hyphal growth did not visually increase from 6 to 9 dpi in the three compatible interactions. The percentages of reached stomata and penetration attempts also remained almost stable at 6 and 9 dpi. Epiphytic development could not be assessed in the compatible interactions after 9 dpi due to massive infectious growth and degradation of the host tissues.

The percentage of successful penetration events was calculated by counting the number of stomata, with a penetration attempt on their ostiole and with infectious hyphae in their sub-stomatal cavities relative to the total number of stomata (Figure 2E and Supplementary Figure 3D). At 4 dpi, infectious hyphae were observed in the sub-stomatal cavities of 3% ± 1% of stomata. At 6 dpi, hyphae successfully penetrated 12% ± 1% of stomata. By 9 dpi, this percentage reached 22% ± 6%, which showed that almost all penetration attempts resulted in a successful penetration event (Figure 2D). These penetration events led to primary sub-stomatal cavities colonization, i.e., colonization from penetrating/primary infectious hyphae.

From the successful penetration events, secondary infectious hyphae colonized the mesophyll following two patterns. The first pattern relied on an extensive growth of branched hyphae around plant cells. The second pattern relied on the formation of straight hyphae, almost not branched, called runner hyphae (Rohel et al., 2001; Shetty et al., 2003; Haueisen et al., 2019). These linear runner hyphae grew longitudinally between the epidermis and the mesophyll, up to 1 cm from the primarily colonized sub-stomatal cavity and branched at regular intervals. The secondary infectious hyphae invaded novel sub-stomatal cavities (i.e., secondary colonization) and colonized deeper in the mesophyll apoplast. To evaluate mesophyll colonization, the percentage of secondary sub-stomatal cavities colonization was quantified (Supplementary Figure 3E). By 9 dpi, 61% ± 3% of sub-stomatal cavities were infected by secondary infectious hyphae (Figure 2G). Since 22% of sub-stomatal cavities were colonized by penetrating/primary infectious hyphae, a total of 83% of sub-stomatal cavities were colonized at 9 dpi, reflecting massive mesophyll colonization. These results, therefore, indicated that, at 9 dpi, a single penetration event leads to the colonization of 3.8 sub-stomatal cavities.

Asexual reproduction was initiated by penetration/primary or secondary infectious hyphae, forming internal rings inside sub-stomatal cavities and aggregating to further develop into young pycnidia. These young pycnidia filled up the sub-stomatal cavities and ultimately matured by growing in size and differentiating pycnidiospores expelled by the stomata ostiole. We quantified (i) the percentage of early-stage pycnidia, which is the number of sub-stomatal cavities, containing ring-forming and aggregating hyphae, relative to the total number of stomata (Supplementary Figure 3F) and (ii) the percentage of young/mature pycnidia (Supplementary Figure 3G). These young/mature pycnidia displayed an intense GFP fluorescence halo, reflecting a dense hyphal structure. At 9 dpi, 26% ± 4% of sub-stomatal cavities were filled with early-stage pycnidia (Figure 2H). At 13 dpi, 47% ± 14% of sub-stomatal cavities were filled with early-stage pycnidia (Figure 2H), and 8% ± 4% of sub-stomatal cavities carried young or mature pycnidia (Figure 2I).

In the incompatible IPO9415/NIL^Stb16q^ interaction, the spore germination rate, the percentages of stomata reached by epiphytic hyphae and of penetration attempts were similar to those observed in the three compatible interactions. However, only few successful penetration events were observed up to 13 dpi (2 and 1% of total stomata at 9 and 13 dpi, respectively) (Figures 2D,F). Since 29 and 36% of stomata displayed penetration attempts in the incompatible interactions at 9 and 13 dpi, respectively, this means that only 3–5% of these attempts resulted in a successful penetration. Still, these few penetration events differed from those of compatible interactions since almost all penetrating infectious hyphae were stopped in the sub-stomatal cavity (Figures 2G–I). Overall, we only observed a single event, over 304 analyzed, in which a penetrating hypha grew in the apoplast and colonized a few neighboring sub-stomatal cavities, without initiating pycnidia formation. These results showed that *Stb16q*-mediated resistance arrests the avirulent isolate during stomata penetration but also most likely prevents hyphae mesophyll colonization from the few successful penetration events.

### *Stb16q* Induces an Early and Transient Stomatal Closure in Response to Avirulent *Zymoseptoria tritici*

**FIGURE 3:**
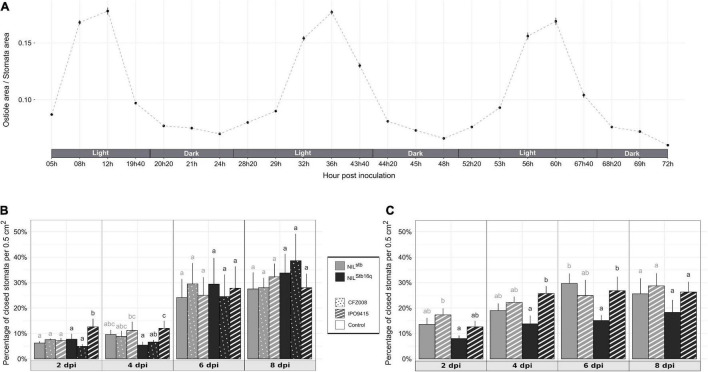
Behavior of wheat stomata in different conditions. **(A)** Stomatal aperture (the ratio of ostiole area/stomata area) calculated from the second leaves of non-inoculated NIL^Stb16q^ along 3 consecutive days. **(B,C)** Percentage of closed stomata of NIL^stb^ (gray bars) and NIL^Stb16q^ (black bars) brush-inoculated with control solution [water/Tween-20, 0.05% (v/v); empty bars], the CFZ008 virulent (dotted bars) or the IPO9415 avirulent (stripped bars) *Z. tritici* isolates in a Conviron^®^ growth chamber **(B)** or in a different growth chamber **(C)**. Values are means ± SEM [*n* = 2 for **(A)**; *n* = 5 for **(B,C)**]. Statistical analyses were performed independently for each day. Different letters indicate significantly different values (ART ANOVA, *p* < 0.05).

We investigated whether *Stb16q* induces stomatal closure, a well-known resistance mechanism limiting pathogens penetration into plant tissues (Grimmer et al., 2012). Stomatal aperture variations of NIL^Stb16q^ were assessed along three consecutive days to identify the most relevant time to study *Stb16q* impact on stomatal closure. Stomatal opening follows a circadian cycle with a maximal opening at midday (Figure 3A). Based on this observation, the percentage of closed stomata (Supplementary Figure 4) for both *Stb16q* NILs inoculated with isolates IPO9415 and CFZ008 was then calculated at 2, 4, 6, and 8 dpi at the maximum stomatal opening time. There were no significant differences in the percentages of closed stomata between mock-inoculated NILs, between the *Z. tritici*- and mock-inoculated NIL^stb^ or between the CFZ008- and mock-inoculated NIL^Stb16q^ at any time points (Figure 3B). By contrast, at 2 and 4 dpi, the percentages of closed stomata were significantly 1.6 to 2.6-fold higher in the IPO9415/NIL^Stb16q^ in compatible interaction compared to CFZ008/NIL^Stb16q^ and mock/NIL^Stb16q^ interactions. As stomatal opening is highly dependent on environmental signals (Fan et al., 2004), the percentage of closed stomata on *Stb16q* NILs inoculated with the avirulent IPO9415 isolate was assessed again in a growth chamber equipped with different lights and a different humidity control system. The variations in the percentages of closed stomata were almost similar to the previous experiment, i.e., an early stomatal closure in the incompatible interaction (1.8-fold higher) compared to the mock/NIL^Stb16q^ interaction, but with a 2-day delay (at 4 and 6 dpi) (Figure 3C). In conclusion, these data demonstrated that *Stb16q* triggers a temporary stomatal closure only when challenged by an avirulent isolate.

### Fusicoccin Does Not Elicit Stomatal Opening on NIL^Stb16q^ Upon Infection With Avirulent *Zymoseptoria tritici*

**FIGURE 4:**
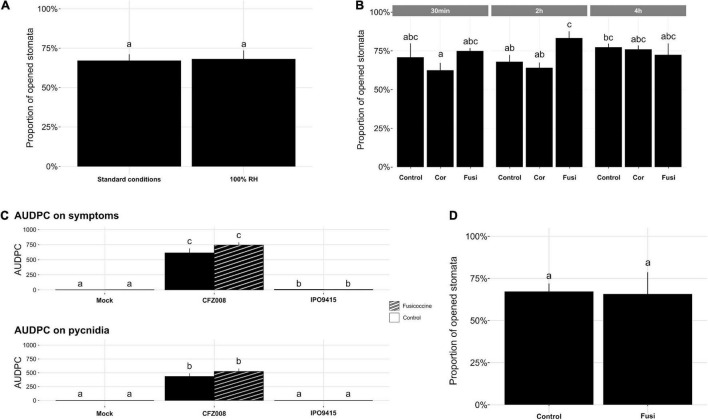
Impact of 100% relative humidity (RH) and toxins on NIL^Stb16q^ stomatal aperture and disease development. **(A)** Percentage of opened stomata on the second leaf of non-inoculated 14-day-old NIL^Stb16q^ grown 3 days at 100% RH, followed by 4 days at 80% RH (standard conditions) or 7 days at 100% RH. **(B)** Percentage of opened stomata on the second leaf of non-inoculated NIL^Stb16q^, 30 min, 2 h or 4 h after being sprayed with water/4% DMSO (v/v) (Control), coronatine (Cor) or fusicoccin (Fusi). **(C)** Symptoms (chlorosis and necrosis) and pycnidia evaluated as the area under disease progression curve (AUDPC) from the second leaves of NIL^Stb16q^ brush-inoculated with control solution [water/Tween-20, 0.05% (v/v), Mock], the CFZ008 virulent or the IPO9415 avirulent *Z. tritici* isolates and sprayed twice at 4-h interval at 4 dpi, with water/4% DMSO (v/v) (empty bars) or with fusicoccin (stripped bars). **(D)** Percentage of opened stomata on the second leaves of NIL^Stb16q^ brush-inoculated with the *Z. tritici* IPO9415 isolate and 2 h after the second spray of water/4% DMSO (v/v) (Control) or fusicoccin (Fusi). Values are means ± SEM [*n* = 4 for **(A,C)**; *n* = 3 for **(B,D)**]. Different letters indicate significantly different values (ART ANOVA, *p* < 0.05).

To study the role of stomatal closure in *Stb16q*-mediated resistance, we investigated whether artificial stomatal opening could promote a bypass of *Stb16q*-mediated resistance by an avirulent isolate. To this end, two strategies were tested. First, 14-day-old NIL^Stb16q^ was grown at 100% relative humidity (RH), a condition known to attenuate stomatal closing in many species (Fanourakis et al., 2020). Second, NIL^Stb16q^ was sprayed with coronatine or fusicoccin that are bacterial and fungal toxins known to force stomatal opening, respectively, in *A. thaliana* and in several species, including durum wheat (Turner and Graniti, 1969; Ricciardi et al., 2003; Melotto et al., 2006). Seven days after 100% RH atmosphere, stomatal behavior of NIL^Stb16q^ was similar to our standard conditions (3 days at 100% RH, followed by 4 days at 80% RH) (Figure 4A), most likely because RH in our standard condition was already quite high. Among toxins, only fusicoccin promoted significantly stomatal opening, since 15% more stomata were opened compared to the control (Figure 4B). This effect was transient and only observed at 2 h post application. To evaluate the impact of this artificial opening on *Stb16q*-mediated resistance, fusicoccin was sprayed on NIL^Stb16q^ inoculated with CFZ008 or IPO9415 isolates. The spray was made twice with 4-h interval at 4 dpi, which corresponded to the beginning of the penetration stage in *Stb16q*-compatible interactions (Figure 2D). Treatment with fusicoccin alone on non-inoculated leaves did not induce foliar symptoms. Treatments of *Z. tritici*-inoculated leaves with fusicoccin did not modify the outcome of the infection as symptoms were similar between control and fusicoccin-treated leaves (Figure 4C). Similar results were obtained when spraying fusicoccin at other kinetic points of the infection (once at 2 or 4 or 6 dpi and once per day every 2 days between 2 and 6 dpi—data not shown). These experiments showed that fusicoccin did not allow avirulent *Z. tritici* to bypass the *Stb16q*-mediated resistance mechanism. In parallel, the effect of fusicoccin on stomatal opening of IPO9415-inoculated NIL^Stb16q^ was evaluated at 4 dpi, 2 h after the second spraying. The proportion of opened stomata was similar between control and fusicoccin-treated leaves (Figure 4D). Overall, our results indicated that, while promoting transient stomatal opening on non-inoculated NIL^Stb16q^, fusicoccin was not able to open stomata of NIL^Stb16q^ infected with avirulent isolate.

### Infiltrated Avirulent *Zymoseptoria tritici* Partially Bypasses *Stb6* and *Stb9*-Mediated Resistances

**FIGURE 5:**
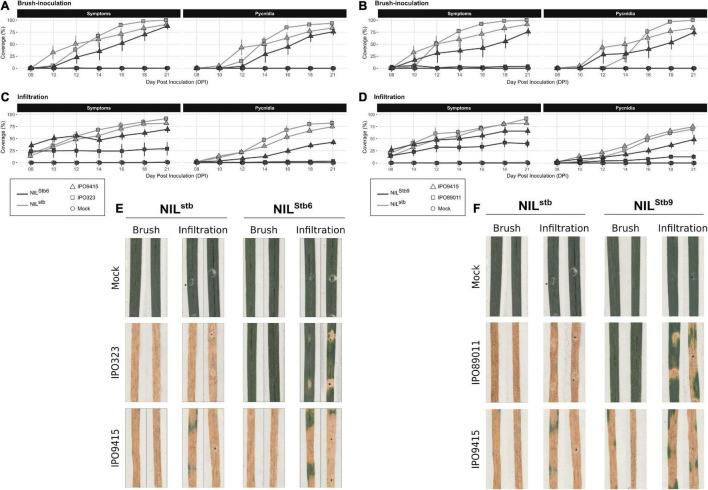
Evolution of symptoms (chlorosis + necrosis) and regions, bearing pycnidia of NIL^stb^, NIL^Stb6^, and NIL^Stb9^ using brush-inoculation **(A,B)** or leaf infiltration **(C,D)**. **(A,C,E)** NIL*^stb^* (gray) and NIL^Stb6^ (black) inoculated with control solution [water/Tween-20, 0.05% (v/v); = Mock, circles], the IPO323 avirulent (squares) or the IPO9415 virulent (triangles) *Z. tritici* isolates. **(B,D,F)** NIL^stb^ (gray) and NIL^Stb9^ (black) inoculated with control solution (= Mock, circles), the IPO89011 avirulent (squares) or the IPO9415 virulent (triangles) *Z. tritici* isolates. **(E,F)** Images of two representative second leaves at 21 dpi. Values are means ± SEM (*n* = 6).

Brush-inoculations and infiltrations of avirulent (IPO323 and IPO89011) and virulent (IPO9415) isolates were performed on *Stb6* and *Stb9* NILs. Following brush-inoculation, symptoms and pycnidia appeared at 10 and 12 dpi, respectively, in all the compatible interactions, and gradually extended up to full coverage of the inoculated leaf area at 21 dpi (Figures 5A,B). With the infiltration method, symptoms and pycnidia appeared at 8 and 10 dpi, respectively, and extended gradually over the infiltrated area, covering 50–75% of the leaf at 21 dpi in all the compatible interactions (Figures 5C,D). In the incompatible IPO323/NIL^Stb6^ and IPO89011/NIL^Stb9^ interactions, no symptoms nor pycnidia were observed at any time point when spores were applied by brush-inoculation (Figures 5A,B). By contrast, when using the infiltration method, symptoms and pycnidia were observed at 8 and 16 dpi, respectively, in the incompatible IPO323/NIL^Stb6^ interaction (Figure 5C). In the infiltrated incompatible IPO89011/NIL^Stb9^ interaction, symptoms and pycnidia were observed at 8 and 12 dpi, respectively (Figure 5D). In both incompatible interactions, symptoms and pycnidia did not exceed 40 and 13% of leaves coverage, respectively, which roughly corresponds to the infiltration spots (Figures 5E,F). In conclusion, these results indicated that, similar to *Stb16q*, avirulent isolates partially bypass *Stb6* and *Stb9*-mediated resistances when infiltrated.

## Discussion

### Quantitative Cytological Analysis of the *Zymoseptoria tritici* Infection Process

The *Z. tritici* infection cycle has been mostly studied using qualitative or semi-quantitative cytological analyses (Cohen and Eyal, 1993; Kema, 1996; Duncan and Howard, 2000; Rohel et al., 2001; Shetty et al., 2003; Jing et al., 2008; Siah et al., 2010; Steinberg, 2015; Fones et al., 2017; Haueisen et al., 2019; Fantozzi et al., 2021). Here, we presented a quantitative cytological analysis over the entire infection process. In agreement with previous observations, we showed that penetration is a continuous and slow process as it occurs between 4 and 9 dpi, increasing in intensity with 25% of successful penetrations (relative to the number of stomata observed) reached at 9 dpi. To compare our results with previous studies, we calculated the penetration efficiency as the number of stomata with successful penetration events relative to the number of germinated spores. The penetration efficiencies in this study varied from 1% ± 1% at 4 dpi to 7% ± 2% at 6 dpi. Due to a dense hyphal network on the leaf surface, the number of germinated spores could not be assessed at 9 dpi. Assuming that this number has reached a maximum by 6 dpi, as reported before (Shetty et al., 2003; Siah et al., 2010; Fones et al., 2017), we estimated an efficiency of penetration of 14% at 9 dpi. Previously, Siah et al. (2010) have shown that *Z. tritici* penetrates into leaf through stomata from 2 dpi, with an efficiency of 7%, to 10 dpi, with a maximum penetration efficiency of 25%. In the study of Shetty et al. (2003) penetration of *Z. tritici* into leaves only started at 5 dpi with a 3.5% efficiency, reaching 6% by 7 dpi. In the study of Fones et al. (2017) few successful penetration events were observed at 2 dpi, but penetration mainly started after a 10-day epiphytic growth, with an efficiency increasing from 4% at 10 dpi up to 40% by 12 dpi. While kinetics and penetration efficiencies are quite similar between the two first studies and our experiment, the study of Fones et al. (2017) differs noticeably, likely reflecting differences in experimental conditions, as proposed by the authors. In other plant pathogenic fungi entering into leaves through stomata, the penetration occurs between 1 and 6 dpi, with efficiencies ranging from 27 to 85% (Yirgou and Caldwell, 1963; Stubbs and Plotnikova, 1972; Kochman and Brown, 1976; Lazarovits and Higgins, 1976; De Wit, 1977; Liu and Harder, 1996). While most of these fungal pathogens develop specialized penetrating structures, known as appressoria, which probably speed up and enhance penetration efficiency, *Cladosporium fulvum*, which lacks appressoria, penetrates into tomato leaves from 4 dpi, with a 30% efficiency, to 6 dpi, reaching 50–70% in efficiency (Lazarovits and Higgins, 1976; De Wit, 1977). In this regard, with a maximum penetration efficiency of 14% by 9 dpi, *Z. tritici* penetration strategy appears slow and relatively underperforming.

Duncan and Howard (2000) observed that, after penetration, some infectious hyphae originating from a single penetration event colonized multiple sub-stomatal cavities (Duncan and Howard, 2000). Here, we provided quantitative evidence of this observation, as we showed that 3.8 sub-stomatal cavities, on average, are colonized by infectious hyphae originating from one penetration site. This massive mesophyll colonization observed from 6 to 9 dpi is consistent with the exponential increase in fungal biomass starting at 6 dpi (Supplementary Figure 1A). These results suggest that the low penetration efficiency of *Z. tritici* might be counterbalanced by a very high efficiency of apoplastic and sub-stomatal cavities colonization. The long *Z. tritici* runner hyphae (up to 1 cm), which were already described (Rohel et al., 2001; Shetty et al., 2003; Haueisen et al., 2019), may play a substantial role in this efficient colonization.

### Penetration Through Stomata: A Critical Stage for *Stb*-Mediated Resistances

We have previously reported that *Stb16q*-mediated resistance stops the avirulent *Z. tritici* isolate 3D7 early in the infection process (Saintenac et al., 2021). Here, we provided quantitative evidences that epiphytic development of the avirulent isolate IPO9415, from germination to penetration attempt, is not affected by *Stb16q* up to 9 dpi. By contrast, we showed that the avirulent isolate is specifically blocked during the penetration stage through stomata when inoculated on NIL^Stb16q^. While almost 100% of the penetration attempts were successful by 9 dpi in all the compatible interactions, in the incompatible IPO9415/NIL^Stb16q^ interaction, only few penetration attempts (3–5%) were successful up to 13 dpi. These infectious hyphae did not further colonize the leaf. In addition, we demonstrated that leaf infiltration of avirulent isolate IPO9415 partially bypasses *Stb16q*-mediated resistance. Our results revealed that *Stb16q*-mediated resistance is mainly acting on avirulent isolates during stomata-mediated penetration. Furthermore, we demonstrated that leaf infiltration of avirulent isolates also partially bypasses *Stb6*- and *Stb9*-mediated resistances. This suggests that blocking the fungus during its penetration into leaf may be a common mechanism in *Stb*-mediated resistances.

Previous cytological studies have compared the *Z. tritici* infection process on resistant and susceptible wheat cultivars (Cohen and Eyal, 1993; Kema, 1996; Shetty et al., 2003; Jing et al., 2008; Siah et al., 2010). In line with our observations, similar germination rates and epiphytic development were reported between compatible and incompatible interactions (Cohen and Eyal, 1993; Kema, 1996; Shetty et al., 2003; Jing et al., 2008; Siah et al., 2010), which suggest that these stages are not affected by wheat resistance mechanisms. In the most comprehensive study, Jing et al. (2008) reported three arrest patterns in the incompatible interactions between 24 *Triticum monococcum* accessions and 9 *Z. tritici* isolates. The first pattern (B) corresponded to infectious hyphae stopped in-between collapsed mesophyll cells. The second pattern (C) corresponded to an arrest of hyphal growth in the intercellular space adjacent to the mesophyll cells surrounding the penetration site. In the third pattern (D), hyphae were stopped immediately after stomata penetration and did not develop in the corresponding sub-stomatal cavity. According to our results, the arrest of the avirulent isolate on NIL^Stb16q^ is very similar to Pattern D described by Jing et al. (2008).

The genetic and molecular nature of the wheat resistances analyzed in these previous cytological studies is unknown. Therefore, it is not possible to link cytological observations and arrest stages of avirulent isolates with *Stb*- or QTL-mediated resistances as we did for *Stb16q*. A parallel between cytological observations of *Z. tritici* infection and macroscopic symptoms can still be drawn. Kema (1996) reported that the intermediate resistance (40% necrosis and 5% pycnidia) of cv. “Shafir” to the IPO235 isolate involved more mesophyll colonization, i.e., Pattern B, than the more-resistant response (5% necrosis and 1% pycnidia) of cv. “Kavkaz/K4500 L6.A.4” to the IPO87016 isolate, which was characterized by hyphae occasionally seen in the vicinity of sub-stomatal cavities, i.e., Pattern C. Studies of Shetty et al. (2003) and Siah et al. (2010) showed that avirulent isolates IPO323 and T0372 were stopped at the penetration site or in its vicinity, i.e., Pattern C, during infection of the resistant cv. “Stakado” and cv. “Scorpion,” respectively. These two cultivars showed strong resistances as infected leaves did not display any visible pycnidia, but only few chloroses. Altogether, these data emphasize the importance of quantitative cytological studies for categorizing wheat resistance mechanisms to STB. Furthermore, these studies suggest that major resistances result primarily in the arrest of the pathogen during penetration (*Stb16q*, Pattern D, and, possibly, *Stb6* and *Stb9*) or during early mesophyll colonization (Kavkaz, Stakado and Scorpion, Pattern C), while more quantitative resistances seem to block the pathogen later on during mesophyll colonization (Shafir, Pattern B).

### Mechanisms Involved in *Stb16q*-Mediated Resistance

As natural openings, stomata are essential for the penetration of many pathogens (bacteria, fungi, and oomycetes) in plant tissues. To prevent pathogens invasion and sporulation, plants have deployed stomatal immunity, a defense mechanism that includes guard cell death and stomatal closure (Chen and Howlett, 1996; Grimmer et al., 2012; Pujol et al., 2016; David et al., 2019; Nogueira Júnior et al., 2020; Ye et al., 2020; Yang et al., 2021). For instance, the dicotyledonous species *Arabidopsis thaliana* and *Vitis vinifera* induce stomatal closure in response to infection by the bacteria *Pseudomonas syringae* and by the oomycete *Plasmoparaviticola*, respectively (Melotto et al., 2017; Nogueira Júnior et al., 2020). At CRKs, the best characterized CRKs, so far, have been associated with stomatal immunity as some of them modulate stomatal aperture in response to bacterial invasion (Bourdais et al., 2015; Yeh et al., 2015; Lee et al., 2017; Kimura et al., 2020). In this context, we investigated the role of stomatal closure in *Stb16q*-mediated resistance and observed a transient stomatal closure specific to the incompatible interaction. However, the similar degree of stomatal closure of this incompatible interaction compared to the compatible interactions on NIL^stb^ at 4 dpi and our failure to artificially open stomata during the incompatible interaction preclude any conclusions on the role of the *Stb16q*-triggered stomatal closure in the arrest of *Z. tritici* at the stomata.

Although the dental resin is a powerful tool to assess stomatal aperture, we were unable to detect fungal hyphae on captured images. Data presented herein are, therefore, a global assessment of all stomata, including those without a penetration attempt. Our cytological assay revealed that 25% of stomata were subjected to a penetration attempt in the incompatible interaction. However, this experiment was performed with a three-time more concentrated inoculum than in the stomatal assay. Assuming a possible linear relationship between penetration attempts and inoculum concentration, we hypothesize that only 8% of stomata were subjected to penetration attempts in our stomatal assay. This percentage is consistent with the percentage of increase in stomatal closure in the incompatible interaction, which suggests an *Stb16q*-dependent localized stomatal closure in response to penetrating hyphae. This hypothesis deserves more investigation but requires optimized techniques to visualize both stomatal aperture and fungal hyphae simultaneously.

Guard cells are highly specialized cells integrating complex environmental signals to modulate stomatal aperture (David et al., 2019). Guard cell perception of the external signals occurs mainly *via* plasma membrane-localized receptor-like protein kinases. For instance, the RLK FLS2 recognizes a conserved domain of the bacterial flagellin. This recognition triggers stomatal closure within 1–2 h, which is dependent of the BAK1 and BIK1 kinases (David et al., 2019). The *Arabidopsis* SIF2 malectin-like receptor-like kinase promotes stomatal closure in response to bacterial infection (Chan et al., 2020). By contrast, the *Arabidopsis* lectin receptor kinase LecRK-V.5 negatively regulates stomatal closure upon *Pst DC3000* infection. Interestingly, these three RLKs are overexpressed in the guard cells, following pathogens infection (Desclos-Theveniau et al., 2012; Beck et al., 2014; Chan et al., 2020). The expression and tissue localization of the plasma membrane-associated STB16 protein is still unknown, but these data question whether it follows the same pattern as these RLKs from *Arabidopsis*, which will reinforce the hypothetical role of *Stb16q* in stomatal immunity.

As proposed by Ye et al. (2020) guard cell death, ultimately leading to stomatal closure, is among the mechanisms underlying stomatal immunity to limit pathogen invasion. During the non-host resistance response of *Brassica juncea* to *Leptosphaeria maculans*, an autofluorescence and a rapid collapse of guard cells were associated with the arrest of avirulent isolates at the stomata (Chen and Howlett, 1996). Similarly, the infection of resistant wheat carrying the 7AL locus by *Puccinia graminis* f. sp. *tritici* resulted in guard cell-localized autofluorescence and Trypan blue coloration, suggesting that the resistance conferred by the 7AL locus involves guard cell death (Pujol et al., 2016). In the *Z. tritici*/wheat pathosystem, stomata cell death and autofluorescence have been reported during the infection of the resistant wheat cv. “Kavkaz/K4500 L.6.A.4,” but not during the infection of the susceptible wheat cv. “Shafir” (Cohen and Eyal, 1993). However, whether these responses were localized at the penetration sites and whether they were involved in resistance were unassessed. Another study reported a localized autofluorescence at 7 dpi for 65% of the stomata subjected to penetration and penetration attempts during the infection of the resistant wheat cv. Stakado (against 2% during the infection of the susceptible wheat cv. Sevin), but without induced cell death (Shetty et al., 2003). Overall, these data and our results emphasize a potential cellular response of stomata during wheat defense against avirulent *Z. tritici* isolates.

Despite a strong arrest at penetration, avirulent *Z. tritici* managed to penetrate 2% of NIL^Stb16q^ stomata. With an average stomatal density of 3,200/cm^2^ on the *Stb16q*NILs, this means that 64 successful penetrations per cm^2^ occurred on NIL^Stb16q^. The infectious hyphae resulting from these successful penetration events were arrested inside or near the infected sub-stomatal cavities. In addition, we observed that the bypass of *Stb16q*-mediated resistance following infiltration of an avirulent isolate is only partial, as symptoms and pycnidia did not extend beyond the infiltrated spots. Together, these results suggest that *Stb16q*-mediated resistance also takes place in the apoplast. Mechanisms involved in restricting *Z. tritici* during incompatible interactions have been suggested, including (i) cell wall reinforcement and remodeling (Cohen and Eyal, 1993; Shetty et al., 2009; Yang et al., 2015; Seybold et al., 2020), (ii) production of PR antifungal proteins (Shetty et al., 2009; Yang et al., 2015), and (iii) generation of a stressful apoplastic environment (Shetty et al., 2003; Yang et al., 2015). In addition, wheat CRK TaCRK3 has been shown to display *in vitro* antifungal activity (Guo et al., 2021; Wu et al., 2021), like other DUF26 containing proteins (Miyakawa et al., 2014; Ma et al., 2018; Guo et al., 2020), suggesting that a direct antifungal effect of *Stb16q* on *Z. tritici* should not be excluded.

## Conclusion

Since little is known about the mechanisms involved in *Stb*-mediated resistance, we intended to identify when and where avirulent isolates were stopped by the *Stb16q* gene. Using wheat unique genetic resources and relevant phenotyping methodologies, we showed that *Stb16q*-mediated resistance mechanism initiates before 7 dpi during the asymptomatic phase. Then, we showed that the avirulent isolate is mainly stopped during the penetration through the stomata as only 5% of penetration attempts were successful compared to almost 100% in the compatible interactions. These penetrating infectious hyphae are not able to colonize the apoplast, suggesting the presence of an *Stb16q*-dependant apoplastic resistant response. In response to infection by the avirulent isolate, a transient stomatal closure was observed only in the wheat NIL carrying *Stb16q*. However, more investigation is needed to determine if a local stomatal closure at the site of fungal penetration could explain the arrest of the avirulent isolate. We further confirmed that penetration is the main arrest stage of the avirulent isolate by *Stb16q* as infiltration of the avirulent isolate directly into wheat leaf apoplast partially bypasses *Stb16q*-mediated resistance. As observed for *Stb16q*, avirulent isolates infiltrated on wheat NILs carrying either the *Stb6* or the *Stb9* genes are able to partially bypass *Stb6* and *Stb9* resistance mechanisms. These results suggest that arrest of the avirulent isolates during stomata penetration might be a shared resistance mechanism mediated by *Stb* genes.

## Data Availability Statement

The original contributions presented in this study are included in the article/Supplementary Material, further inquiries can be directed to the corresponding author/s.

## Author Contributions

TL and CS designed the research. FC and OS developed wheat near-isogenic lines. MB and FC prepared the samples and performed the experimentations. M-HL and KS were involved in the cytological assay, from samples preparation to data analysis. MB and CS analyzed the data, prepared the figures, and wrote the manuscript. M-HL and TL proofread the manuscript. All authors contributed to the article and approved the submitted version.

## Conflict of Interest

The authors declare that the research was conducted in the absence of any commercial or financial relationships that could be construed as a potential conflict of interest.

## Publisher’s Note

All claims expressed in this article are solely those of the authors and do not necessarily represent those of their affiliated organizations, or those of the publisher, the editors and the reviewers. Any product that may be evaluated in this article, or claim that may be made by its manufacturer, is not guaranteed or endorsed by the publisher.
