# Supplementary material for: Blocked at the Stomatal Gate, a Key Step of Wheat Stb16q-Mediated Resistance to Zymoseptoria tritici
Source: Front Plant Sci. 2022 Jun 27;13:921074. doi: 10.3389/fpls.2022.921074 (PMC9271956; doi:10.3389/fpls.2022.921074)
Supplement: Supplementary file 1 [file Data_Sheet_1.PDF]

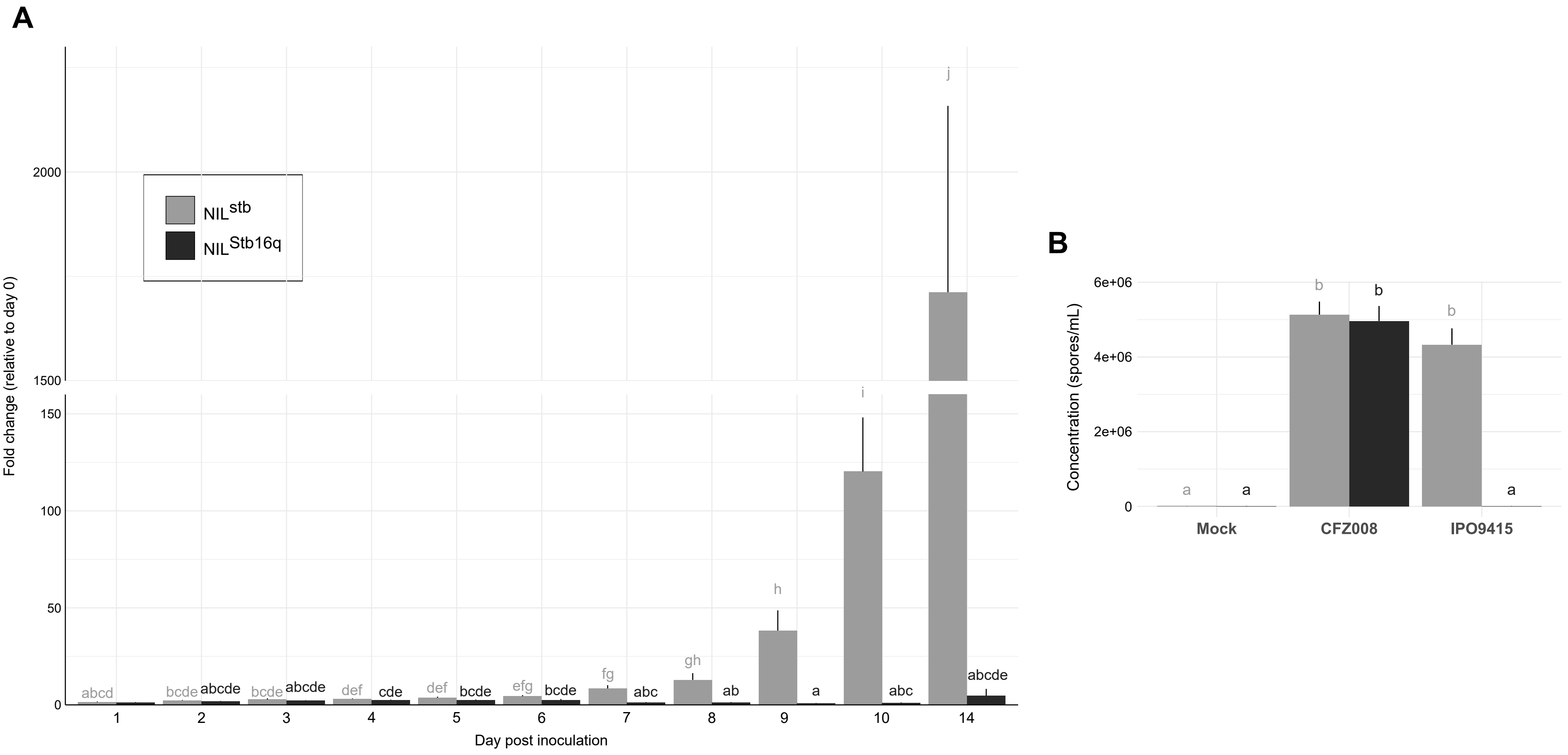

Supplementary Figure 1. *Z. tritici* biomass and sporulation on NIL^{stb} (grey) and NIL^{Stb16q} (black) after brush-inoculation. **(A)** The IPO9415 avirulent isolate and plant biomasses were measured by RT-qPCR with the IGS and 18S primers, respectively. The relative expression was calculated with the $2^{-\Delta\Delta CT}$ method, using day 0 (just after brush-inoculation) as the reference. **(B)** Spores concentration of the virulent CFZ008 and the avirulent IPO9415 isolates 25 days post inoculation. Values are means ± SEM [n = 10 for **(A)**; n = 9 for **(B)]**. Different letters indicate significantly different values (Linear mixed model for **(A)** and ART anova for **(B),** $p < 0.05$).
