# Supplementary material for: Blocked at the Stomatal Gate, a Key Step of Wheat Stb16q-Mediated Resistance to Zymoseptoria tritici
Source: Front Plant Sci. 2022 Jun 27;13:921074. doi: 10.3389/fpls.2022.921074 (PMC9271956; doi:10.3389/fpls.2022.921074)
Supplement: Supplementary file 2 [file Data_Sheet_2.PDF]

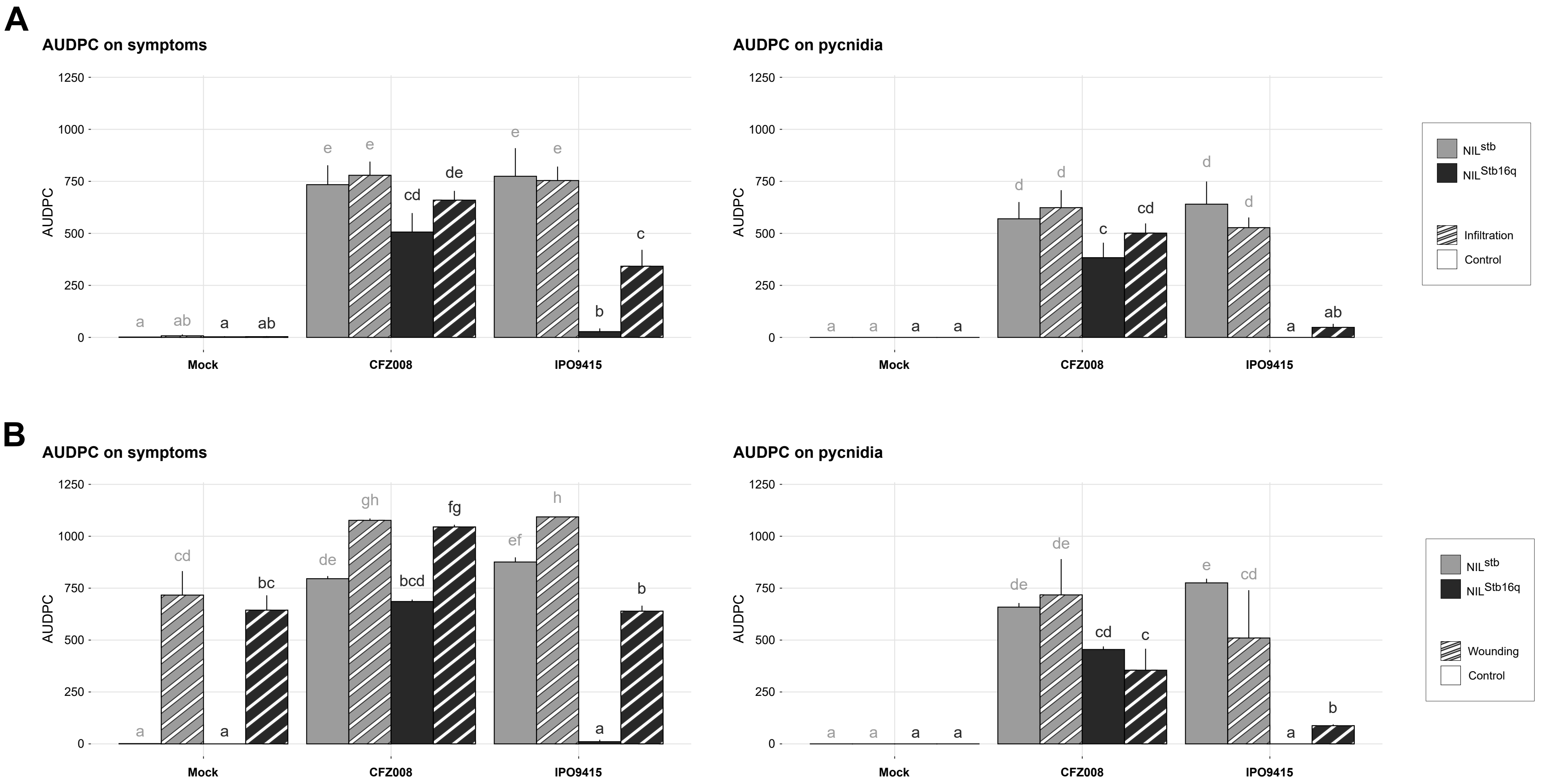

Supplementary Figure 2. Symptoms (chlorosis + necrosis) and pycnidia, evaluated as the Area Under Disease Progression Curve (AUDPC), of NIL^{stb} (grey) and NIL^{Stb16q} (black) inoculated with control solution (water/Tween20 0.05% (v/v); = Mock), the IPO9415 avirulent or the CFZ008 virulent *Z. tritici* isolates and with different inoculation methods. **(A)** Comparison between brush-inoculation (empty bars) and infiltration (stripped bars) methods. **(B)** Comparison of the brush-inoculation method on unwounded (empty bars) or wounded (stripped bars) leaves. Values are means ± SEM [n = 8 for **(A)**; n = 3 for **(B)**]. Different letters indicate significantly different values (ART anova, $p < 0.05$).
