# Supplementary material for: Blocked at the Stomatal Gate, a Key Step of Wheat Stb16q-Mediated Resistance to Zymoseptoria tritici
Source: Front Plant Sci. 2022 Jun 27;13:921074. doi: 10.3389/fpls.2022.921074 (PMC9271956; doi:10.3389/fpls.2022.921074)
Supplement: Supplementary file 3 [file Data_Sheet_3.PDF]

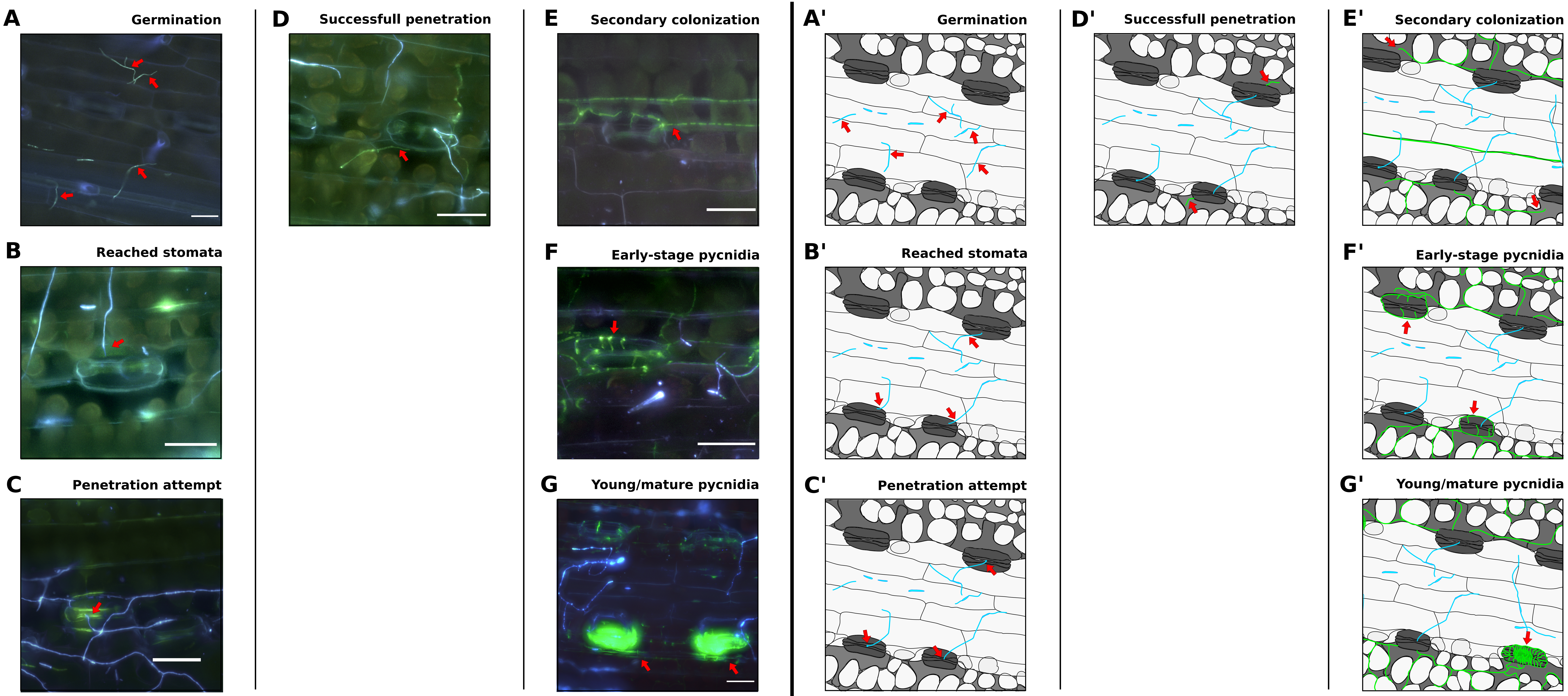


**Supplementary Figure 3.** Illustrations of the different stages of *Z. tritici* infection cycle on *Stb16q* NILs. Spores of virulent GFP-labelled *Z. tritici* isolates were brush-inoculated on wheat second leaves. Leaves were collected at different times post-inoculation and fungus was visualized with the chitin surface-dye calcofluor (blue) to detect external hyphae and with the GFP fluorescence. **(Left panel)** maximum projection of epifluorescence microscopy images of the different stages. Bar = 50 µm. **(Right panel)** schematic representation of the different stages. Red arrows indicate events counted for the quantitative analysis (Figure 2). **(A, A')** Germination (= germinated spores). **(B, B')** Reached stomata (= stomata with hyphae in contact). **(C, C')** Penetration attempt (= stomata with hyphae diving into the ostiole). **(D, D')** Successful penetration (= stomata with hyphae going through the ostiole and in the sub-stomatal cavity). **(E, E')** Secondary sub-stomatal cavities colonisation (= stomata with hyphae only in the sub-stomatal cavity, without hyphae above the ostiole). **(F, F')** Early-stage pycnidia (= stomata with branching and ring-forming hyphae in the sub-stomatal cavity). **(G, G')** Yound and mature pycnidia (= stomata with densified hyphae structure, in the form of GFP halo).
