# Supplementary material for: Blocked at the Stomatal Gate, a Key Step of Wheat Stb16q-Mediated Resistance to Zymoseptoria tritici
Source: Front Plant Sci. 2022 Jun 27;13:921074. doi: 10.3389/fpls.2022.921074 (PMC9271956; doi:10.3389/fpls.2022.921074)
Supplement: Supplementary file 4 [file Data_Sheet_4.PDF]

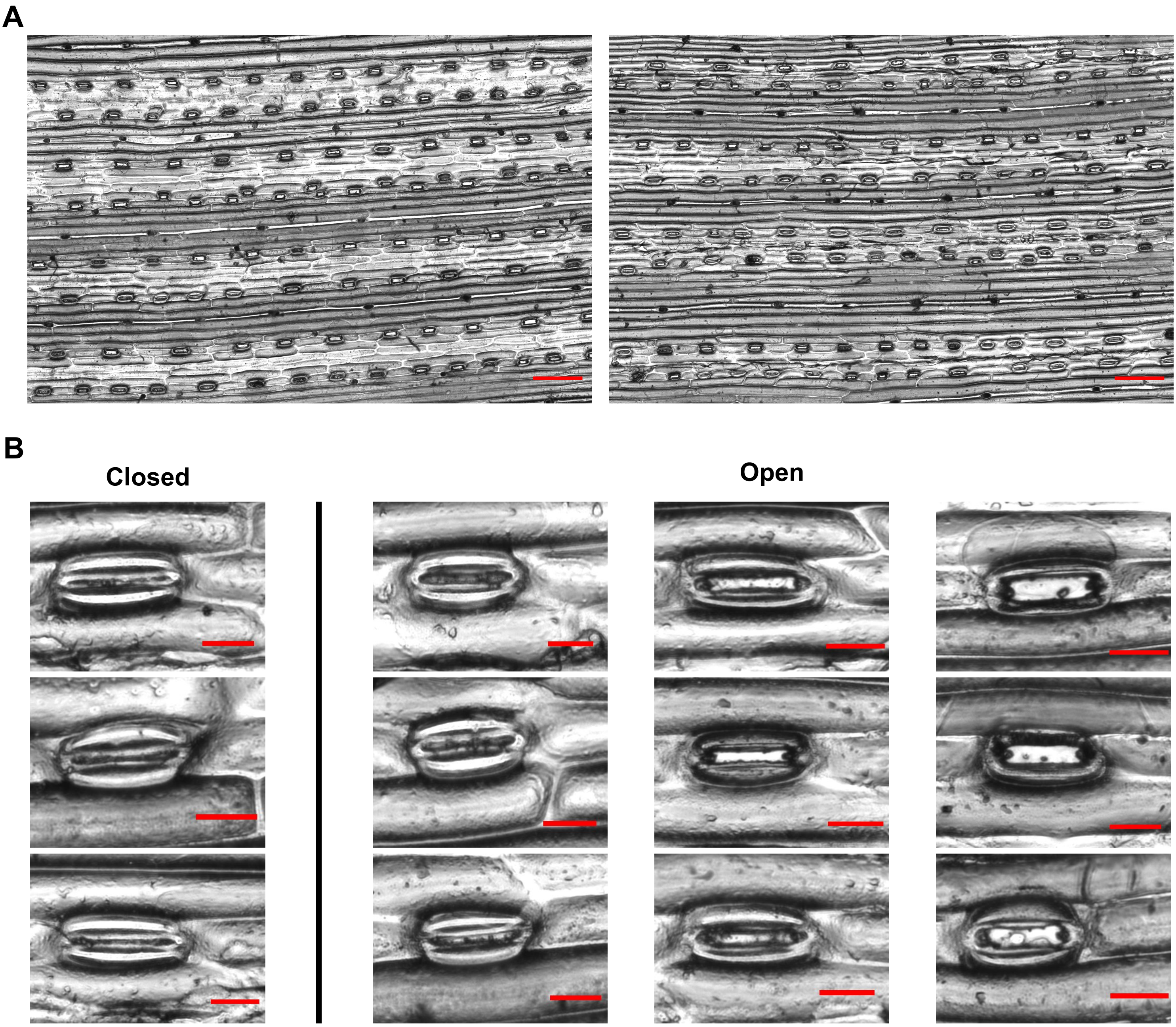

Supplementary Figure 4. Images of stomata from *Stb16q* NILs. Imprints of leaves were made using dental resin. Transparent nail polish was applied on imprints and observed under a light microscope, with phase contrast. **(A)** Representative part of 0.5 cm^2-tiles images. Bar = 200 µm. **(B)** Representative stomata classified as “closed” or “open" to evaluate *Stb16q* and toxins impact on stomatal opening. Bar = 30 µm.
